# Rapid Profiling of the Volatilome of Cooked Meat by PTR-ToF-MS: Characterization of Chicken, Turkey, Pork, Veal and Beef Meat

**DOI:** 10.3390/foods9121776

**Published:** 2020-11-30

**Authors:** Qianlin Ni, Iuliia Khomenko, Luigi Gallo, Franco Biasioli, Giovanni Bittante

**Affiliations:** 1 Department of Agronomy, Food, Natural Resources, Animals, and Environment (DAFNAE), University of Padova, Viale dell’Università 16, 35020 Legnaro (PD), Italy; qianlin.ni@studenti.unipd.it (Q.N.); giovanni.bittante@unipd.it (G.B.); 2 Department of Food Quality and Nutrition, Research and Innovation Centre, Fondazione Edmund Mach (FEM), Via E. Mach 1, 38010 San Michele all’Adige (TN), Italy; iuliia.khomenko@fmach.it (I.K.); franco.biasioli@fmach.it (F.B.)

**Keywords:** cooked meat odor, cooked meat flavor, flavor fingerprint, volatile organic compounds

## Abstract

This study aimed to compare the volatile organic compound (VOC) profiles of cooked meat from different species. Four burgers were prepared and cooked from each of 100 meat samples obtained from 100 animals of five species/categories (chicken, turkey, pork, veal and beef) sourced from five supermarkets and five local butchers. Two burgers were cooked in a water bath and two were grilled. Direct proton-transfer-reaction time-of-flight mass-spectrometry (PTR-ToF-MS) analysis of the sample headspace yielded 129 mass peaks, 64 of which were tentatively identified. The results showed that turkey and chicken had the largest and the smallest total concentrations of all VOCs, respectively. Of the mammalian meats, veal and beef had greater total VOC concentrations than pork. The proportions of the amounts of all the individual VOCs differed significantly according to species. Additionally, 14 of 17 independent latent explanatory factors (LEFs) identified by multivariate analysis exhibited significant differences between meat species/categories, and therefore helped to characterize them. PTR-ToF-MS has been used for the first time for the rapid and non-invasive profiling of cooked meat of different species/categories. Knowledge of specific VOC profiles paves new avenues for research aimed at characterizing species through sensory description, at authenticating species or at identifying abnormalities or fraud.

## 1. Introduction

Flavor is a fundamental attribute in meat evaluation. During and after the cooking process, in particular, a complex series of chemical reactions between precursors, intermediate reaction products and degradation products results in many compounds in the meat being modified or generated and possibly dispersed in air [1]. These volatile organic compounds (VOCs) are the key constituents of meat odor and flavor and drive the perception of meat quality [2] and customer acceptability [3].

The content and nature of the VOC precursors in meat and of the VOCs that develop after cooking [4] are affected by several factors, including genetics (species, breed, selection, sex), feeding, animal management and meat processing and storage [1]. The majority of studies on non-cured meat VOCs have been carried out on chicken and beef: Lytou et al. [5] identified 50 VOCs and Zhou et al. [6] 33 VOCs in chicken meat, whereas Saraiva et al. [7] detected 54 VOCs in raw beef, and Watanabe et al. [8] detected 70 VOCs in cooked beef. Serrano et al. [9] investigated the VOC profile of meat from young bulls and identified 2,3-octanedione, skatole and terpenes as biomarkers of grass feeding. Park et al. [10] investigated VOC induced by fatty acid oxidation in pork meat. Brunton et al. [11] found six VOCs related to pentanal and hexanal and Mielnik et al. [12] found 20 VOCs in turkey meat; Gkarane et al. [13] identified 63 VOCs in lamb meat. However, most of the studies on meat VOCs have focused on the metabolism pathways of the volatile compounds, including some off-flavors.

The effects of the species of origin on the VOC profiles of meat have been scarcely investigated. Comparisons based on studies carried out on meat from a single species may be affected by differences in meat origin, meat sampling procedures, and analytical methods and instruments employed. On the other hand, only few studies have aimed to compare and characterize the VOC profiles of meat originated from different species [14]. Therefore, there is large room for improving our knowledge about the differences on VOC profiles and characteristics of meat obtained by the main species used for meat production for human consumption. Indeed, accurate comparison of the volatile profiles of different types of meat is important as a proof of concept for a more in-depth analysis of meat, and to support product development and combat counterfeiting and fraud. It is difficult to compare the VOCs of meat from different species based on published results because studies often deal only with single meat types, which means any differences found could be related to the different origins and processing procedures of the meat samples, or to different analytical approaches.

Thus, the main aim of this study was to determine and compare using the same analytical procedure, the VOC profiles of a selection of the most common meat types (chicken, turkey, pork, veal and beef) sourced from several representative suppliers and processed using the same procedures. Specific aims were: (i) to build a collection of meat cuts representing the major species/categories and a variety of suppliers and conditions; (ii) to characterize through a large number of VOCs the volatile organic profiles of the cooked meat samples using a direct, non-invasive, direct injection mass spectrometry technique, namely, proton-transfer-reaction time-of-flight mass-spectrometry (PTR-Tof-MS); (iii) to identify and compare the VOC profiles and the latent explanatory factors (LEFs) characterizing cooked meat from the five most important species and categories (chicken, turkey, pork, veal, and beef).

## 2. Materials and Methods

### 2.1. Experimental Design

A project was set up with the aim of studying the VOC profiles of meats from different species and cooked using different methods. A parallel study within the same project dealt with the relationships among meat VOCs, and with identifying and characterizing the independent LEFs underlying the phenotypic measurements of individual VOCs [15]. In accordance with the project’s objective, we adopted a hierarchical experimental design with the following structure:

- Compare the 5 most important species/categories of meat: chicken, turkey, pork, veal and beef;
- Obtain simultaneously samples of all the species from each of a wide variety of retailers (10 sampling sessions: 5 supermarkets belonging to different major chains and 5 local butchers);
- Account for variation between animals within retailers by sampling 2 animals per species/category per session (5 species × 10 suppliers/sessions × 2 animals = 100 animals sampled, 20 per species/category);
- Compare two very different cooking methods for each animal sampled: rapid grilling on a high-temperature surface, and slow cooking at a moderate temperature;
- Account for variation among meat samples within animal and cooking method by preparing two burgers per animal and cooking method (100 animals × 2 cooking methods × 2 burgers/method = 400 burgers).
  No live animals were used for the research and therefore no ethical authorization was needed.

### 2.2. Meat Sampling

Meat samples were collected and prepared over 10 consecutive weekly sessions. In each session meat samples of all the species/category of concern were purchased from a different retailer. The breast muscles (pectoralis major and minor) of the avian species, and the loin muscles (longissimus lumborum) of the mammalian species (pork, veal and beef) were sampled, to obtain a cut of about 600–800 g per animal per session (2 animals per each species/category, 10 cuts per session). Packaged meat cuts were obtained from the self-service refrigerated meat displays of the supermarkets (5 sessions), whereas the meats from the local butchers (5 sessions) were freshly cut and packaged according to local practice. All retailers were regularly monitored by the local public health agency, and each cut was aged according to local customs (about two weeks for beef, a few days for the other species/categories). The samples were kept in transportable refrigerators at 4 °C, and shortly after collection were transferred to the refrigerators of the DAFNAE meat laboratory of the University of Padova (Padova, Italy).

### 2.3. Raw Meat Processing and Cooking

Physical analyses of the fresh meat, the sampling of meat aliquots for chemical analyses, grinding of meat samples, preparation and cooking of burgers, physical analyses of the cooked burgers and sampling of aliquots of the cooked burgers for VOCs and other chemical analysis were carried out on all samples in a given session the day after they were obtained from the retailer.

Each meat sample was trimmed to remove excess fat, cartilage and any intermuscular membranes, and cut into about 2cm-sized cubes. The cubes were minced in a commercial meat grinder (FAMA TS12 meat mincer, FAMA Industrie, Rimini, Italy) in the order pork, veal, beef, turkey, chicken, with the tools cleaned between each sample to avoid cross-contamination. Four burgers (meat patties) of around 110 g each (1.1 cm thickness) were formed from each minced meat sample using a hand-held hamburger press (FAMA FHA100, FAMA Industrie, Rimini, Italy). Patties were weighed and then cooked in two different ways: 2 burgers from each meat sample (2 burgers × 2 animals × 5 species/categories = 20 burgers per session) were sealed in polyethylene bags and cooked in a water bath preheated at 75 °C to an internal temperature of 70 °C [16]. The other two patties from each meat sample were dry-heat cooked on electric griddle (Tristar grill BP 2970), at 163 °C for 3 min per side, until a target internal temperature of 70 °C [17].

### 2.4. Analyses of the Characteristics of Fresh and Cooked Meats

pH was measured on all the fresh meat samples, using a Sension+ pH-meter (HACH, Milano, Italy) equipped with a glass 5053T electrocode suitable for meat penetration and an automatic temperature compensator. Before analysis, the pH-meter was calibrated using standard buffers (pH 4.0 and 7.0). Color was measured using a Minolta CM-600d spectrophotometer (Konica Minolta Sensing Americas, Inc.; Ramsey, NJ, USA) on the surface of each patty. The instrument was calibrated on its own white reference tile supplied by the manufacturer and set with the illuminant D65, which represents average daylight. CIELAB coordinates lightness (L*), redness (a*) and yellowness (b*) were recorded. Three random readings were taken at different locations on the patty surface and averaged.

An aliquot of each minced meat sample was delivered to chemical laboratory of the DAFNAE for analyzing chemical composition according to [18]. Namely, moisture was determined by leaving overnight in an oven at 101–103 °C (method 950.46); crude protein (CP) was measured by multiplying the organic N content by 6.25 (method 976.05); fat was determined by extraction with petrol ether (method 991.36); and ash was determined by mineralization in a muffle furnace at 550 °C (method 920.153).

Color traits were assessed on the cooked surface of each patty after one-hour cooling at room temperature using the same procedures described above for raw patties. Thereafter, each cooked patty was tested for shear force measured on one strip (2.5 cm wide) removed from the center across the width from each patty. The strip was sheared (crosshead speed of 250 mm/min) perpendicular to the cooked surface using a multi-bladed Allo-Kramer shearing device equipped with 10 blades attached to a Lloyd (Bognor Regis, UK) LS5 AMETEK testing machine equipped with NEXYGEN PLUS 3 software. Shear force values were recorded in N/g.

After cooking, 3 g of meat were taken from the center of each burger and placed into glass vials (20 mL, Supelco, Bellefonte, PA, USA), capped with PTFE/Silicone septa (Supelco) and stored at −80 °C until VOC analysis.

### 2.5. Analysis of the Volatile Organic Compounds

The volatilome of each of the cooked meats was analyzed following the procedure described in detail in a parallel study within the same project [15]. In brief, the head spaces of the cooked meat samples were analyzed directly with a proton-transfer-reaction time-of-flight mass-spectrometer (PTR-ToF-MS 8000; Ionicon Analytik GmbH, Innsbruck, Austria) coupled with a multipurpose GC automatic sampler (Autosampler, Gerstel GmbH, Mulheim am Ruhr, Germany) at the Food Quality and Nutrition Department of Edmund Mach Foundation (San Michele all’Adige, Trento, Italy). Briefly, the vials were thawed at room temperature for 6 h. For each batch, a maximum of 124 samples were chosen randomly from the 400 cooked meat samples and measured on the same day. The vials were kept at 4 °C prior to analysis, and each vial was incubated for 20 min at 25 °C immediately before analysis. The instrumental conditions in the drift tube were as follows: drift voltage 628 V, drift temperature 110 °C, drift pressure 2.80 mbar, affording an E/N value of about 130 Townsend (1 Td = 10–17 cm^2^/V·s), where E corresponds to the electric field strength and N to the gas number density. The sampling time per channel of ToF acquisition was 0.1 ns, amounting to 350,000 channels for a mass spectrum ranging up to *m*/*z* = 350. Every single spectrum is the sum of 28,600 acquisitions of 35 μs each, resulting in a time resolution of 1 s. Sample measurement was performed in 70 cycles resulting in an analysis time of 60s/sample. A 4-min interval was kept between measurements to avoid memory effects. As detailed in Bittante et al. [15], after spectral analysis, peaks were extracted from the aligned spectra and their amplitude converted in ppbv (part per billion by volume).

A total of 383 mass peaks were extracted from the raw data, and after the routine mass peak selection procedure (elimination of mass peaks related to isotopologues and internal ions produced by PTR-ToF-MS, selection of mass peaks with concentrations from meat samples significantly different from blank samples) 129 mass peaks were selected for further analysis. The descriptive statistics of these PTR-ToF-MS mass peaks are presented and discussed in the parallel study [15]. The sum of the areas of all 129 peaks was calculated and constitutes the “quantitative” data of each burger analyzed; the areas of the 129 peaks expressed as proportions of their sum constitute the “qualitative” data (or profile) of each burger analyzed. In the following sections of this article, the mass peaks are tentatively identified based on sum formula and literature data, even though it is not possible to exclude completely the interference of different isomeric molecules or fragments.

### 2.6. Data Editing and Univariate Statistical Analysis of the Sources of Variation in VOCs

Meat burger samples with abnormal VOC profiles were identified on the basis of Mahalanobis distance [19]: samples with Mahalanobis distances outside the interval mean ± 3.0 SD were considered sample outliers and all their VOC contents were discarded (11 out of 400 samples in quantitative dataset and 14 out of 400 samples in qualitative dataset). 

The data were analyzed using PROC MIXED (SAS Institute Inc., Cary, NC, USA) according to the following hierarchical mixed model:

y_ijklmn_ = µ + RT_i_ + s_j_ + SC_k_ + a_l_ + CM_m_+ (SC × CM)_km_ + e_ijklmn_ (1)

where y_ijklmn_ was the observed variable (the sum of all VOCs and the 129 individual peaks expressed as a proportion of their sum); μ is the overall mean; RT_i_ is the fixed effect of the i-th retailer type (i = 1, 2); s_j_ is the random effect of the j-th session/retailer within each retailer type (j = 1, …, 10); SC_k_ is the fixed effect of the k-th species/category within session (k = 1, …, 5); a_l_ is the random effect of the l-th animal within species/category (l = 1, …, 100); CM_m_ is the fixed effect of the m-th cooking method within animal (m = 1, 2); (SC × CM)_km_ is the fixed effect of the km-th interaction between species/category and cooking method (km = 1, …, 10); and e_ijklmn_ is the random residual term ≈ N(0, σ^2^).

After a first run with this model, individual VOCs with concentrations outside the interval mean ± 3.0 RSD were considered VOC outliers and their values were omitted from the analyses of the individual VOCs, although the corresponding meat samples were retained with all the other VOCs. After this editing procedure, the model was run again on the resulting dataset and these results are presented in this study. The same editing procedure and univariate analysis were used to process the sum of all the VOCs (quantitative trait).

In accordance with the hierarchical design adopted, the fixed effect of retailer type (RT_i_) was tested using the variance of random effect of session/retailer within retailer type (s_j_) as the error line. The fixed effect of meat species/categories (SC_k_) was tested using the variance of the random effect of animal (al) as the error line. Lastly, the fixed effects of cooking method (CM_m_) and of the (SC × CM)_km_ interaction were tested using the variance of random residual (e_ijklmn_) as the error line. 

The 4 degrees of freedom of the fixed effect of meat species/category (C = chicken, T = turkey, P = pork, V = veal and B = beef) were analyzed through the following orthogonal contrasts:

- Comparison of classes: poultry (C + T) vs. mammals (P + V + B);
- Comparison of species within poultry: C vs. T;
- Comparison of species within mammals: P vs. (V + B);
- Comparison of categories within cattle species: V vs. B.

Sourcing meat samples from 10 different retailers of two different types (supermarkets and local butchers) ensured that a range of suppliers was represented and that the comparisons among the 5 meat species/categories were not biased by having been obtained from a single source. As the effect of retailer type is not an objective of this study, it is not presented nor discussed here. In any case, the effect was very seldom significant, and as the experimental design was balanced none of the results presented is biased by this effect.

Including two cooking methods in the study allowed us to make comparisons among the 5 species/categories with respect to different meat cooking procedures frequently used in research dealing with meat quality assessment [17]. Not being an objective of this study, the effects of cooking methods and their interactions with meat species/category will not be presented nor discussed here. Although the effect of cooking method was almost always highly significant, and the interaction between meat species/category and cooking method was significant for most of the VOCs and LEFs, it was rarely so large as to change the ranking among meat species/categories. For this reason, the main effects of meat species/category presented here may be considered representative across cooking methods.

### 2.7. Multivariate Analyses of Latent Explanatory Factors of VOCs

The values of burger meat samples for the 129 mass peaks of the VOC profiles correlated with each other in various directions and with various strengths. A detailed description of these analyses can be found in the parallel study, and as latent explanatory factor analysis was the object of this study, details of the methodology used and interpretation of the results are also given [15]. Briefly, the factor analysis was conducted using SAS PROC FACTOR (SAS 9.4) with the Varimax rotation, and yielded 17 LEFs. The eigenvalues of the factors and the communality values for the measured variables after rotation were also obtained. 

The LEFs obtained from this dataset were:

- LEF-1, explaining 35.1% of total variance, is based on 48 significant VOCs (having a loading >+0.5 or <−0.5) out of 129; only 2 of these were quantitatively more relevant (>10 µg/L): *m*/*z* 33.034 (tentatively identified (t.i.) as methanol), and *m*/*z* 61.035 (t.i. acetic acid, and fragment of acetate esters as butyl acetate, 2-methylbutyl acetate, isobutyl acetate);
- LEF-2, explaining 20.8% of total variance, is based on 28 significant VOCs, of which the relevant ones are *m*/*z* 53.039 and *m*/*z* 69.070 (t.i. pentanal, pentenol);
- LEF-3, explaining 9.5% of total variance, is based on 13 significant VOCs, of which the relevant ones are *m*/*z* 41.038 (common fragment), *m*/*z* 43.055 (common fragment), *m*/*z* 53.003, *m*/*z* 57.034 (t.i. propenal), *m*/*z* 57.070 (t.i. butanol, isobutanol) and *m*/*z* 71.085 (t.i. methyl butanol, pentanol);
- LEF-4, explaining 7.6% of total variance, is based on 7 significant VOCs, of which the relevant ones are *m*/*z* 43,018 (common fragment) and *m*/*z* 89.060 (t.i. acetoin (3-hydroxy-2-butanone), ethyl acetate, butanoic acid);
- LEF-5, explaining 4.7% of total variance, is based on 5 significant but quantitatively not relevant VOCs;
- LEF-6, explaining 4.4% of total variance, is based on 7 significant but not relevant VOCs;
- LEF-7, explaining 4.4% of total variance, is based on 5 significant VOCs, one of which is quantitatively relevant *m*/*z* 75.944;
- LEF-8, explaining 3.8% of total variance, is based on 4 significant but not relevant VOCs;
- LEF-9, explaining 3.5% of total variance, is based on 4 significant but not relevant VOCs;
- LEF-10, explaining 3.4% of total variance, is based on 4 significant VOCs, of which *m*/*z* 31.019 (t.i. formaldehyde) and *m*/*z* 60.053 (acetone isotopolougue) are relevant;
- LEF-11, explaining 3.1% of total variance, is based on 4 significant VOCs, of which the relevant ones are *m*/*z* 46.996 (t.i. thioformaldehyde), *m*/*z* 49.008 (methanethiol) and *m*/*z* 55.050 (t.i. butanal);
- Another 6 minor LEFs (LEF-12 to LEF-17) explain between 2.9 and 1.6% of total variance: one (LEF-13) is based on 2 VOCs, one of which is relevant (*m*/*z* 63.026, t.i. dimethyl sulfide, ethanthiol), 4 (LEF-12, LEF-14, LEF-15, and LEF-16) are based on one significant VOC and one (LEF-17) had no VOCs reaching the threshold.

The scores of each burger sample for each of the 17 LEFs were then analyzed using the same univariate hierarchical mixed model previously described for individual VOCs.

## 3. Results

Descriptive statistics of some selected meat traits are summarized by species/category in Table 1.

The results obtained from the entire dataset of the flavor profiles (129 VOCs) of the 400 samples from the cooked patties are presented and discussed in a parallel study [15], which also reports the results of the multivariate analysis by which the 17 LEFs underlying the phenotypic measures of the VOCs were identified. We report and discuss here the results obtained comparing the flavor profiles of the cooked patties made from meat from the five species/categories analyzed.

**Figure 1:**
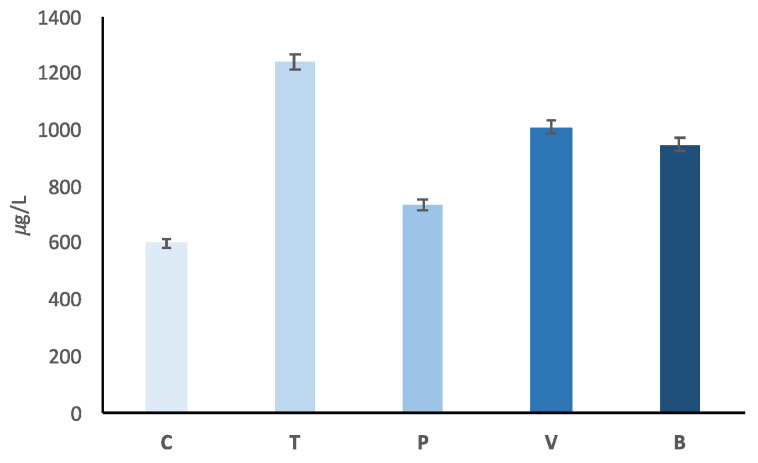
Sums of concentrations (µg/L) of 129 volatile mass peaks in the head spaces of cooked meat samples; results are given with standard error. The 2 avian breast meats (chicken—C and turkey—T) are not significantly different from the 3 mammalian loin meats (pork—P, veal—V and beef—B); chicken meat had a lower (*p* < 0.001) concentration than turkey meat; pork had a lower (*p* < 0.001) concentration than bovine (veal and beef) meats; veal and beef meats were not significantly different (RMSE: 183 µg/L).

The analysis of variance showed that the total concentration of the volatile compounds (sum of all VOCs) did not, on average, differ in the cooked patties made from avian breast and those made from mammals’ loin cuts (Figure 1). Within poultry species, however, the total concentration in turkey meat was about double than that in chicken meat, and within mammals it was much greater in bovine than in porcine species. We observed no difference between the two bovine categories of meat (veal and beef).

As proportions of the sum of all VOCs, all the individual VOCs were affected by species/category for at least one of the four orthogonal contrasts conducted (Table 2). 

**Figure 2:**
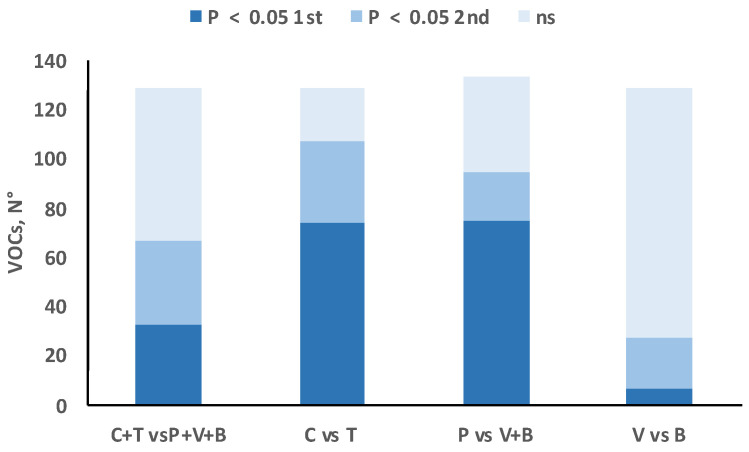
Number of volatile organic compounds (VOCs) with significant differences in favor of the first vs. second term of the contrasts (*p* < 0.05 1st), in favor of the second vs. first term of the contrasts (*p* < 0.05 2nd) and with no significant differences between the 2 terms of the contrasts (ns) for each of the four orthogonal contrasts tested (C = chicken; T = turkey; P = pork; V = veal; and B = beef meat).

Moreover, about half of the 129 individual VOCs were present in significantly different proportions in the meats from avian and mammalian species; nearly 80% and 70% of VOCs were present in significantly different proportions in the meats from chickens and turkeys and in those from pigs and bovines, respectively, whereas differences in proportions of individual VOCs between meats from beef and veal were close to only 20% (Figure 2).

Only 3 of the 17 LEFs extracted from the VOC database (LEF-3, LEF-5 and LEF-9) presented no significant differences among the meat samples of the five species/categories examined here (Table 3).

**Figure 3:**
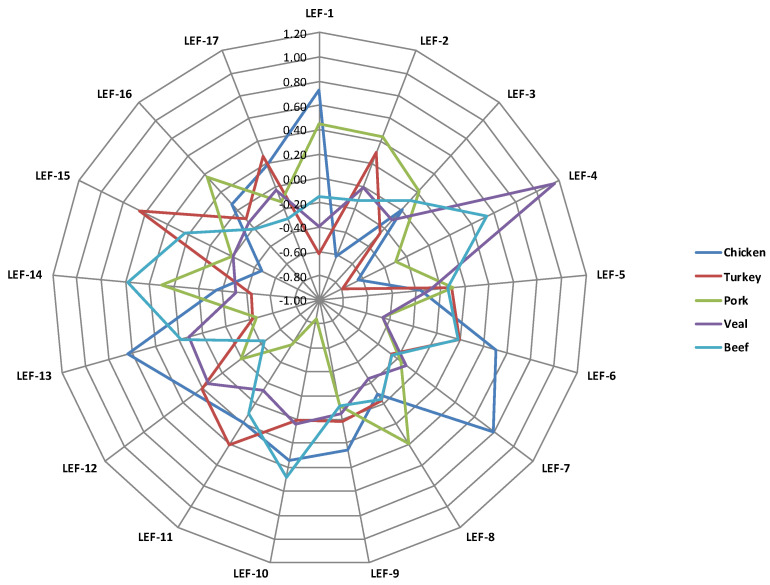
Radar graph plotting the least square means of latent explanatory factor (LEF) scores of meat VOCs according to different animal species.

Therefore, least square means of LEF scores of meat VOCs (Figure 3) mostly differentiated the meat samples originated by different species/categories of animals.

## 4. Discussion

PTR-ToF-MS has been shown to be a powerful tool for directly and rapidly characterizing the volatilomes of foods [19,20]. Due to the very high number of individual VOCs found in the headspaces of the cooked meat samples in the vials, and the numerous and complex relationships among them, summarizing them and describing the flavors of the cooked meat using these data were very difficult tasks that required tailored statistical tools.

The authors are unaware of any information in the scientific literature regarding the total amounts of VOCs in meat from the main animal species/categories. The uniqueness of the data reported here lies also in the fact that the meat samples from different species/categories were always collected, processed and analyzed together by the same personnel/laboratory/instruments/methods. Moreover, by collecting meat samples of the five species/categories from 10 different suppliers of two different types of retailers (supermarket chains vs. local butcheries), we were able to reproduce in the experimental dataset a large part of the variability often found in practice. The variability among different meat suppliers is due to differences in the animals’ breeds/crossbred genotypes, sexes and production systems; age at slaughter and slaughter procedures, in the meat processing and aging procedures; and in sales practices. Lastly, by cooking all meat samples according to two very different procedures we were also able to obtain average figures that were not specific to a particular cooking method. The effects of any cooking method and its interaction with species/category are beyond the aims of this study, and will be dealt with in another specific analysis. In any case, it is worth pointing out that, even though the interaction between cooking method and species/category of meat was often statistically significant (also because of the large number of samples analyzed), it was very seldom relevant from a practical point of view and did not alter the ranking of the species/categories for the different VOCs and their LEFs.

The results show the effect of species/category of slaughtered animals to be very important, evidenced by some significant differences in all the individual VOCs and 14 of the 17 LEFs among the cooked meat samples from the five species/categories. In line with the hierarchical structure of the experimental design, the five species/categories were not compared through multiple unstructured comparisons (all vs. all), but rather through four orthogonal contrasts (equal to the number of degrees of freedom available) that hierarchically compared, firstly, the two classes of animals (avian vs. mammal); then, the two species within each class (chicken vs. turkey, and pig vs. cattle); and lastly, the two meat categories within the bovine species (veal vs. beef). The discussion will follow that same order.

### 4.1. Flavor Profiles of Cooked Meat from Avian vs. Mammal Species

The first orthogonal contrast compared the breast meat from the two avian species (chicken and turkey) with the loin meat from the three mammalian species/categories (pork, veal and beef). 

As seen in Figure 1, from a quantitative perspective, the average of the sum of all VOCs analyzed in poultry species did not differ significantly from the average obtained from mammal meat. 

From a qualitative point of view, the situation is very different because, even though their summed concentrations are similar, there are significant differences between the two classes of animals with respect to many of the individual VOCs expressed as proportions of their sum (Table 2). As summarized in Figure 2, about half of the 129 individual VOCs were present in significantly different proportions in the meats from avian and mammalian species, and half were present in similar proportions. Of the former, about the same number of VOCs were present in greater proportions in avian species as were present in greater proportions in mammalian species. Discussion of all 129 individual VOCs is beyond the scope of this study, but the LEFs made it possible to largely reduce the dimensionality of the dataset. Table 3 shows that about half the LEFs (9 out of a total of 17) differed significantly between the average of the two avian species and the average of the three mammalian species, yet none of them had positive least square means (LSM) of the scores for all the species of a given animal class and negative LSMs for all the species of the other class, the only exception being the least important LEF (LEF-17, explaining less than 2% of total covariance). This means that there is no single LEF that could be used to clearly discriminate between poultry meat and mammalian meat burgers. The differences between the different species within each class were much greater than the differences between the two classes of species. We have found no previous research comparing the VOC profiles of meat from at least two species of either the mammal or poultry class, so specific literature comparisons cannot be made.

### 4.2. Flavor Profiles of Chicken vs. Turkey Cooked Meat

Within avian species, we found a very large difference in the overall concentrations of VOCs: the concentration of all VOCs in turkey meat was about double that in chicken (Figure 1). From a quantitative point of view, these two species presented the extreme LSM values among all the five species/categories studied. From a qualitative point of view, the cooked patties made from chicken and turkey meat had very different flavors: 107 of the 129 individual VOCs were present in significantly different proportions in the two avian species (Table 2). The greater number of these VOCs (74) were present in larger proportions in the chicken patties, while only 33 were present in larger proportions in the turkey patties (Figure 2). As these values are proportions of a sum, the imbalance in the numbers is due to the fact that a few major VOCs in greater proportions in one species are offset by many minor VOCs in greater proportions in the other. In fact, some of the VOCs present in greater amounts were found in greater proportions in cooked turkey meat patties than in chicken meat patties: 41.038 *m*/*z* (common fragment), 49.008 *m*/*z* (t.i. as methanethiol), 55.050 *m*/*z* (t.i. butanal) and 101.097 *m*/*z* (hexanal, hexan-1-one, hexan-2-one). Chicken is probably the most widely studied of all species for meat flavor [1,21], much more so than turkey, but we are unaware of any direct comparisons of the two having been made.

Even though 83% of the individual VOCs were present in significantly different proportions in the cooked patties of the two avian species, only 5 of the 17 LEFs differed significantly (Table 3). These, however, include the two most important ones, which together represent 75 of the 129 VOCs and about 56% of all covariance among the individual VOCs. The radar graph (Figure 3) plotting the least square means of the LEF scores of the meat VOCs illustrates the comparison between the two avian species.

In the case of the most important LEF (LEF-1), the two poultry species presented the two extreme values among all five species/categories studied: the chicken patties had the highest (positive) scores, and the turkey patties the lowest (negative) scores. The meat LEFs are reported and discussed in the parallel study [15]; nonetheless, here and in the following paragraphs we will briefly summarize them to provide a better understanding of their meaning relative to the effects of species/category of animal. It is worth noting that LEF-1 groups 48 of the 129 VOCs and summarizes 35% of the variability among all VOCs. Two of these VOCs are present in average concentrations >10 µg/L and can therefore be considered as having high quantitative relevance for meat flavor: *m*/*z* 33.034 (t.i. methanol) and *m*/*z* 61.035 (t.i. acetic acid, fragment of butyl acetate, 2-methylbutyl acetate, isobutyl acetate). Both these mass peaks, and other mass peaks characterizing LEF-1—*m*/*z* 95.019 (t.i. dimethyl sulfone), *m*/*z* 103.048, *m*/*z* 115.079, *m*/*z* 117.092 (t.i. hexanoic acid, ethyl butanoate, methyl isovalerate), and *m*/*z* 121.066 (t.i. acetophenone, 4-methyl-benzaldehyde)—have been detected in chicken by [22,23,24,25].

No sensory evaluation is available for characterizing the individual LEFs, but because of the number of VOCs involved, LEF-1 probably represents the basic “meaty” flavor of cooked meat. The parallel study also suggests that, based on the known odor of the more intense peaks, LEF-1 could be characterized by fresh, fruity, pungent and garlic odors. 

LEF-2 is, by definition, independent of LEF-1, and here the situation is reversed: turkey patties have much higher scores than chicken patties (Figure 3). LEF-2 is also very important, explaining 21% of total covariance and based on 28 of the 129 volatile mass peaks, of which those that could be considered relevant are *m*/*z* 53.039 and *m*/*z* 69.070 (t.i. pentanal, pentenol). Based on the quantitatively most relevant VOCs, LEF-2 could be characterized by green, leafy, nutty, waxy and fruity odors. The volatilome of turkey meat has not been greatly studied, although Brunton et al. [11] identified *m*/*z* 67.055 on cooked turkey meat. 

LEF-7 also differed in the two poultry species, and as is clear from Figure 3, this LEF could be considered specific to cooked chicken patties (the only ones with positive LSM scores). It is of less importance, grouping seven VOCs (4.4% of total covariance), but unfortunately these VOCs have not yet been clearly characterized in terms of odor (unknown odor). One of the peaks belonging to this LEF, *m*/*z* 59.967 (unidentified), has been identified in chicken by [26], giving a fruity flavor. Lastly, LEF-13 identifies chicken patties, and LEF-15 turkey patties, but these LEFs have very low importance and their odors have not been characterized.

### 4.3. Flavor Profile of Pork vs. Bovine Cooked Meat

From a quantitative point of view (Figure 1), there were far fewer VOCs in cooked pork patties than in bovine (veal and beef) meat patties. Qualitatively, 91 of the 129 individual VOCs were present in different proportions in the two mammalian species (Table 2 and Figure 2). Here, too, there was an imbalance between the two species, as the number of VOCs with higher proportions in pork than in bovine patties (75) was much greater than the number of VOCs with higher proportions in bovine than in pork patties (only 16). Although the most prevalent of all the VOCs (*m*/*z* 55.050, t.i. butanal) was more abundant in cooked pork patties, the second (*m*/*z* 43.018, common fragment) and the fourth (*m*/*z* 49.008, t.i. methanethiol) were more abundant in the bovine patties (Table 2). Moreover, the proportion of *m*/*z* 63.026 (t.i. dimethyl sulfide, ethanthiol), the ninth VOC in terms of concentration in cooked meat, in bovine patties was about 10 times that in pork patties.

Almost half (8 out of 17) of the LEFs were present in different proportions in the cooked patties made from meat of the two mammalian species (Table 3). As clearly shown in Figure 3, LEF-1 and LEF-2, previously discussed in comparing chicken and turkey meats, were more abundant in porcine than in bovine patties. In contrast, LEF-4 was characteristic of bovine meats, as not only was it proportionally much more abundant in them than in pork; it was even more abundant in them than in both avian species (Figure 3). LEF-4 explained 7% of total covariance and was characterized by seven VOCs, of which the relevant ones were *m*/*z* 43,018 (common fragment) and *m*/*z* 89.060 (t.i. acetoin (3-hydroxy-2-butanone), ethyl acetate, butanoic acid). On the basis of the known odors associated with some of the constituents, this LEF is thought to characterize “malty, butter and roast odors.” LEF-16 is based on only one VOC.

More specific to cooked pork patties (also vs. avian meats) were LEF-8 and LEF-16. LEF-8 explained almost 4% of total covariance and is characterized by 4 VOCs, none of which could be considered quantitatively relevant; these may confer mainly fruity notes to meat. LEF-16 is based on only one VOC (*m*/*z* 79.039), which could be considered a water cluster of C_2_H_4_O_2_H^+^.

LEF-10, on the other hand, displayed reversed specificity for cooked pork patties, with very low values compared not only with bovine, but also with all species. This LEF explained 3.4% of total variance, and was also characterized by 4 VOCs, two of which could be considered quantitatively relevant for meat flavor: *m*/*z* 31.019 (t.i. formaldehyde), and *m*/*z* 60.053 (isotope of t.i. acetone, propan-2-one), tending to confer pungent, irritating odors. 

Similarly, LEF-11 and LEF-13 displayed significantly lower values for pork compared with bovine patties. They explain 3.1% and 2.5% of total covariance, and are characterized by four and two VOCs, respectively. LEF-11 is probably very important for meat flavor, as three of the four VOCs could be considered quantitatively important: *m*/*z* 46.996 (t.i. thioformaldehyde), *m*/*z* 49.008 (t.i. methanethiol) and *m*/*z* 55.050 (t.i. butanal), likely conferring acrid, sulfurous, roast odors. The two mass peaks characterizing LEF-13 are *m*/*z* 62.023 (t.i. nitromethane, methyl nitrite) and *m*/*z* 63.026 (t.i. dimethyl sulfide, ethanthiol). Published studies on the VOCs of pork meat products deal much more frequently with preserved meats (sausages, hams, etc.) than with cooked fresh meats [27,28].

### 4.4. Flavor Profile of Pork vs. Bovine Cooked Meat

From a quantitative point of view, the total concentrations of VOCs were similar in the cooked burgers made from the two bovine meat categories (Figure 1). From a qualitative point of view, only 27 VOCs were found in significantly different proportions in the two bovine categories, as shown in Table 2 and Figure 2. In this case, more VOCs characterized beef (20) than veal (only 7), but the only major VOCs affecting the bovine categories were 43.018 *m*/*z* (common fragment) for veal, and *m*/*z* 33.034 (t.i. methanol) and *m*/*z* 73.065 (t.i. 2-butanone, butanal) for beef. Regarding the LEFs, Table 3 shows that six differed in the two bovine categories, despite the small number of individual VOCs differentiating the two bovine categories. The three most important LEFs did not differ between the two bovine categories, but the fourth (LEF-4), which the previous orthogonal contrast showed to be very specific to bovine species (probably conferring malty, butter and roast odors), showed higher scores in cooked veal than in cooked beef patties (Figure 3). LEF-12 also showed higher scores for veal patties (3% of covariance explained, characterized by only one VOC: *m*/*z* 52.028 (unidentified). LEF-12 is not very important, as it is a one-VOC factor (*m*/*z* 52.028, unidentified) and explains less than 3% of total covariance.

We have not found any studies on the VOC profile of veal, as research tends to focus on beef, but our results show that there is less of a difference between the VOC profiles of these two categories than between the VOC profiles of different species.

LEF-6, LEF-10, LEF-15 and especially LEF-14 showed higher scores in beef than in veal burgers. LEF-6 explained 4.4% of total covariance and is based on seven VOCs, none quantitatively relevant, although some of them have been documented in beef and are known for some flavor components. Lustig and Schuetz [29] detected a peak close to *m*/*z* 28.032 on meat and thought it just came from the packaging. Peaks *m*/*z* 111.118 and *m*/*z* 115.113 are related to octanol/octanal and heptanal/heptan-2-one respectively, which have been detected in beef [30,31], and associated with a fruity, fatty, sweet odor [24]. Peak *m*/*z* 143.146 is related to methyloctanol, nonanal and nonan-2-one, which have also been detected in beef [3,32]. Nonanal contributes a fatty, grassy odor, whereas nonan-2-one gives a plastic, earthy odor. Peak *m*/*z* 129.128 (t.i. octanal, octanone) is associated with a mushroom-like odor [24]. Peak *m*/*z* 143.146 (methyloctanol, nonanal, nonan-2-one) has also been detected in beef [3,32], imparting a fatty, grassy, plastic, earthy flavor.

LEF-10, as seen regarding its very low LSM characterizing pork samples, is based on peaks tentatively associated to formaldehyde (*m*/*z* 31.019), acetone and propan-2-one (isotopologue at *m*/*z* 60.053), propylene glycol (*m*/*z* 77.059) and C9 esters and acids (*m*/*z* 159.137), and thus could be related to pungent, irritating notes. LEF-14 is not very important, as it is based on only one VOC (*m*/*z* 109.076, t.i. 2,5-dimethylpyrazine) and explains less than 2% of total covariance. Lastly, LEF-15, despite showing significantly higher scores in beef than in veal meat (Table 3), is not specific to the former, as it is proportionally more abundant in turkey meat (Figure 3).

## 5. Conclusions

From a methodological point of view, our work demonstrates the feasibility of rapid, non-invasive fingerprinting of the meat volatilome, which might be exploited in many different applications. All of the many volatile organic compounds of cooked meat are affected by animal species and category. About half the VOCs and their latent explanatory factors are significantly different in poultry (chicken and turkey) breast meat compared with mammalian (pork, veal and beef) loin meat. The differences are even larger between the two avian species (chicken vs. turkey) and between the two mammalian species (porcine vs. bovine), but are much smaller between the two categories of the bovine species (veal vs. beef). Different latent explanatory factors could be used to investigate the volatilomes of different species/categories, although further study is required for better characterizing their sensory meaning and to investigate the possible interactions with cooking methods.

## Figures and Tables

**Table 1:** Descriptive statistics (means ± SD) of characteristics of the meat sampled before (20 animals per species/category) and after processing and cooking (80 meat patties per species/category).

Items:	Chicken	Turkey	Pork	Veal	Beef
Raw patty color:					
- lightness, L*	49.1 ± 1.8	47.7 ± 2.1	48.1 ± 3.4	49.9 ± 4.5	37.1 ± 4.6
- redness, a*	2.4 ± 1.1	1.1 ± 1.1	3.3 ± 1.8	5.9 ± 2.4	16.0 ± 2.9
- yellowness, b*	18.7 ± 4.0	9.1 ± 1.2	13.4 ± 1.4	14.6 ± 1.9	17.6 ± 2.4
Raw patty composition (g/100 g):					
- moisture	75.3 ± 0.9	73.7 ± 0.8	72.4 ± 0.9	75.2 ± 1.8	73.7 ± 1.0
- protein	21.9 ± 0.7	23.8 ± 0.8	22.5 ± 0.8	20.4 ± 0.9	22.0 ± 0.6
- lipids	1.3 ± 0.4	0.7 ± 0.3	3.3 ± 1.3	2.7 ± 2.4	2.6 ± 1.2
- ash	1.1 ± 0.0	1.1 ± 0.0	1.1 ± 0.0	1.1 ± 0.1	1.1 ± 0.1
Raw patty pH:	6.02 ± 0.08	5.89 ± 0.24	5.68 ± 0.06	5.64 ± 0.10	5.60 ± 0.09
Cooked patty color:					
- lightness, L*	75.8 ± 5.0	71.6 ± 7.4	64.8 ± 8.3	60.2 ± 8.6	50.6 ± 10.4
- redness, a*	3.4 ± 2.3	3.8 ± 3.4	5.4 ± 4.3	5.6 ± 3.5	7.2 ± 3.1
- yellowness, b*	22.2 ± 6.3	19.1 ± 6.5	20.4 ± 6.5	21.2 ± 4.6	20.4 ± 3.6
Cooked patty shear force, N/g	14.6 ± 2.1	19.5 ± 2.8	21.6 ± 3.7	18.5 ± 4.0	25.2 ± 5.6

**Table 2:** Effects of meat species and category on the least square means and the contrast significance of the relative percentage incidence of the area of each volatile mass peak on the sum of all 129 mass peaks of cooked meat patties.

*m/z*	Raw Formula	Tentative Identification	Least Squares Means ^1^					Orthogonal Contrasts (*p* Value)				RMSE ^2^
			C	T	P	V	B	C + T vs. P + V + B	C vs. T	P vs. V + B	V vs. B	%
26.016	C_2_H_2_^+^	Common fragment	0.730	1.011	1.010	0.881	0.895	-	<0.001	<0.001	-	0.162
28.032	C_2_H_4_^+^		0.104	0.094	0.077	0.068	0.084	<0.001	-	-	<0.001	0.020
29.039	C_2_H_5_^+^	Common fragment	0.361	0.228	0.322	0.247	0.244	0.03	<0.0001	<0.0001	-	0.072
29.060			0.028	0.032	0.035	0.029	0.028	-	0.01	<0.0001	-	0.004
31.019	CH_2_OH^+^	Formaldehyde	1.577	1.703	1.202	1.574	1.800	-	-	<0.0001	0.02	0.301
33.034	CH_4_OH^+^	Methanol	3.279	2.244	4.217	3.146	4.230	<0.0001	<0.0001	<0.0001	<0.0001	0.697
34.996	H_2_SH^+^	Hydrogen sulfide	3.870	0.521	0.887	0.635	0.704	<0.0001	<0.0001	-	-	0.603
38.018			0.040	0.047	0.050	0.044	0.046	-	0.02	0.04	-	0.012
41.038	C_3_H_5_^+^	Common fragment	7.874	8.255	9.126	8.174	8.919	0.05	-	-	-	1.632
42.011			0.905	0.965	1.143	0.915	0.966	0.02	-	<0.0001	-	0.198
42.034	C_2_H_3_NH^+^	Acetonitrile	2.290	1.261	1.995	1.430	1.678	-	<0.0001	0.01	-	0.740
43.018	C_2_H_3_O^+^	Common fragment	7.092	5.559	8.824	12.882	11.225	<0.0001	<0.0001	<0.0001	<0.001	1.899
43.055	C_3_H_7_^+^	Common fragment	2.605	1.974	2.902	2.827	2.736	<0.001	0.03	-	-	0.816
46.034	C_13_CH_4_OH^+^		0.570	0.672	0.764	0.735	0.709	<0.0001	<0.0001	0.03	-	0.085
46.996	CH_2_SH^+^	Thioformaldehyde	1.739	2.002	0.586	0.896	0.851	<0.0001	-	-	-	0.991
47.049	C_2_H_6_OH^+^	Ethanol	1.571	0.833	1.165	1.066	0.933	-	<0.001	-	-	0.507
49.008	CH_4_SH^+^	Methanethiol	7.738	14.756	1.846	4.875	4.100	<0.0001	<0.0001	<0.001	-	4.640
52.028			0.006	0.007	0.006	0.005	0.005	<0.0001	-	<0.001	-	0.002
53.003			1.374	1.628	1.708	1.514	1.596	0.05	<0.001	0.03	-	0.259
53.039	C_4_H_5_^+^		1.454	2.902	2.601	2.262	2.075	-	<0.0001	0.01	-	0.595
55.050	C_4_H_7_+	Butanal	14.363	21.819	24.769	19.182	17.166	0.01	<0.0001	<0.0001	-	5.483
57.034	C_3_H_4_OH^+^	Propenal, or common fragment	1.596	1.940	2.074	1.732	1.838	0.04	<0.0001	<0.001	-	0.318
57.070	C_4_H_9_^+^	Butanol, isobutanol	1.877	1.267	1.729	1.300	1.500	-	<0.0001	0.01	-	0.560
59.967			0.275	0.156	0.198	0.122	0.153	<0.0001	<0.0001	<0.001	-	0.061
60.053	C_2_^13^CH_7_^+^	Isotope of Acetone, Propan-2-one	2.247	1.169	0.990	1.550	1.710	<0.0001	<0.0001	<0.0001	-	0.308
61.035	C_2_H_4_O_2_H^+^	Acetic acid, fragment of Butyl acetate, 2-Methylbutyl acetate, Isobutyl acetate	5.513	2.705	5.084	3.793	4.187	-	<0.0001	<0.0001	-	1.615
62.023			0.077	0.025	0.024	0.053	0.052	-	<0.0001	<0.0001	-	0.016
63.026	C_2_H_6_SH^+^	Dimethyl sulfide, ethanthiol	6.067	1.778	0.390	3.648	3.837	0.01	<0.0001	<0.0001	-	1.617
63.947			0.003	0.001	0.001	0.001	0.001	<0.0001	<0.0001	-	-	0.001
63.986			0.078	0.029	0.049	0.037	0.039	<0.0001	<0.0001	<0.0001	-	0.013
67.021			0.011	0.017	0.008	0.009	0.010	<0.0001	<0.0001	-	-	0.005
67.055	C_5_H_7_^+^	Pentenal or common fragment	0.273	0.435	0.419	0.341	0.345	-	<0.0001	<0.001	-	0.067
67.992			0.002	0.002	0.002	0.002	0.002	-	0.02	0.03	-	0.001
69.034	C_4_H_4_OH^+^	Furan	0.058	0.058	0.083	0.060	0.066	0.02	-	<0.001	-	0.049
69.070	C_5_H_9_^+^	Isoprene or common fragment of aldehydes, alcohols and terpenes)	2.006	3.543	3.179	2.389	2.720	-	<0.0001	<0.001	-	0.694
70.004			0.004	0.003	0.004	0.003	0.004	-	<0.001	<0.001	-	0.001
71.015			0.031	0.027	0.033	0.035	0.036	<0.0001	0.01	-	-	0.006
71.049	C_4_H_6_OH^+^	2-Butenal, Methyl vinyl ketone	0.271	0.217	0.421	1.381	0.984	<0.0001	-	<0.0001	<0.0001	0.384
71.085	C_5_H_11_^+^	Methyl butanol, Pentanol	2.062	1.342	2.013	1.822	2.033	0.04	<0.001	-	-	0.711
73.065	C_4_H_8_OH^+^	2-Butanone, Butanal	4.364	2.350	2.385	2.176	4.196	-	<0.0001	0.05	<0.0001	0.970
75.028	C_3_H_6_SH^+^	Allyl mercaptan	0.096	0.049	0.081	0.063	0.067	-	<0.0001	<0.0001	-	0.021
75.044	C_3_H_6_O_2_H^+^	Propanoic acid, Methyl acetate?	0.374	0.251	0.415	0.381	0.382	<0.0001	<0.0001	0.02	-	0.081
75.081	C_4_H_10_OH^+^		0.059	0.035	0.056	0.038	0.042	-	<0.0001	<0.001	-	0.030
75.944			3.676	0.912	1.308	1.023	1.110	<0.0001	<0.0001	-	-	1.424
77.016			0.027	0.016	0.022	0.018	0.020	0.03	<0.0001	<0.001	0.02	0.005
77.059	C_3_H_8_O_2_H^+^	Propylene Glycol	0.892	0.723	0.474	0.742	0.854	<0.0001	0.01	<0.0001	-	0.156
77.976			0.054	0.026	0.030	0.023	0.028	<0.0001	<0.0001	-	-	0.012
78.979			0.006	0.007	0.006	0.005	0.006	<0.001	0.02	0.02	-	0.004
79.039	C_2_H_6_O_3_H^+^	Adduct of water and C_2_H_4_O_2_H^+^	0.149	0.072	0.161	0.118	0.124	<0.0001	<0.0001	<0.0001	-	0.051
79.055	C_6_H_7_^+^	Benzene, Aromatic fragment	0.536	0.447	0.659	0.355	0.477	-	-	0.01	-	0.643
79.938			0.009	0.002	0.003	0.003	0.003	<0.0001	<0.0001	-	-	0.003
80.041			0.016	0.008	0.015	0.011	0.012	-	<0.0001	<0.0001	-	0.003
81.038	C_5_H_4_OH^+^		0.063	0.038	0.054	0.043	0.049	-	<0.0001	<0.0001	0.00	0.009
81.071	C_6_H_9_^+^	Hexenal, common fragment	0.249	0.433	0.389	0.340	0.349	-	<0.0001	0.04	-	0.085
82.047			0.008	0.006	0.007	0.005	0.006	<0.0001	<0.0001	<0.001	0.03	0.002
84.044	C_5_^13^CH_11_^+^	Hexanal, Hexenol	0.019	0.014	0.021	0.014	0.015	-	<0.0001	<0.0001	-	0.006
85.014	C_4_H_4_SH^+^	Thiophene	0.050	0.027	0.026	0.016	0.022	<0.001	<0.001	-	-	0.020
85.073			0.052	0.055	0.059	0.051	0.052	-	-	<0.0001	-	0.009
85.101	C_6_H_13_^+^	Hexanol	0.248	0.170	0.255	0.191	0.218	-	<0.001	0.03	-	0.109
86.022			0.012	0.006	0.009	0.006	0.008	<0.001	<0.0001	<0.0001	0.04	0.003
86.970			0.008	0.005	0.011	0.010	0.010	<0.0001	<0.0001	-	-	0.004
87.044	C_4_H_6_O_2_H^+^	2,3-Butanedione, diacetyl	0.315	0.243	0.394	0.616	0.581	<0.0001	-	<0.0001	-	0.150
87.080	C_5_H_10_OH^+^	Pentanal, Pentanone	0.432	0.756	0.810	0.673	0.741	<0.0001	<0.0001	0.01	-	0.230
88.960			0.007	0.003	0.007	0.010	0.008	<0.0001	<0.0001	<0.001	0.01	0.002
89.060	C_4_H_8_O_2_H^+^	Acetoin (3-Hydroxy-2-butanone),Ethyl acetate, Butanoic acid	0.393	0.235	0.997	3.609	2.302	<0.0001	-	<0.0001	<0.0001	0.726
91.059	C_4_H_10_SH^+^	Diethyl sulfide	0.143	0.096	0.143	0.116	0.121	-	<0.001	0.02	-	0.073
93.069	C_7_H_9_+	Toluene	0.369	0.207	0.273	0.198	0.214	<0.001	<0.0001	0.01	-	0.176
95.019	C_2_H_6_O_2_SH^+^	Dimethyl sulfone	0.410	0.199	0.359	0.265	0.253	-	<0.0001	<0.0001	-	0.139
95.053			0.140	0.086	0.133	0.092	0.105	-	<0.0001	<0.0001	-	0.040
95.088	C_7_H_11_^+^	Heptenal, common fragment	0.062	0.071	0.072	0.062	0.065	-	<0.001	<0.001	-	0.011
97.064	C_6_H_8_OH^+^	2,5-Dimethylfuran, Ethylfuran	0.049	0.091	0.056	0.067	0.078	-	<0.0001	<0.001	0.03	0.021
97.101			0.208	0.176	0.180	0.167	0.228	-	0.02	-	<0.0001	0.048
99.082	C_6_H_10_OH^+^	2-Hexenal, Trans-2-hexenal, 2-Hexanone, Hexanone acid	0.048	0.087	0.077	0.068	0.071	-	<0.0001	-	-	0.019
101.097	C_6_H_12_OH^+^	Hexanal, Hexan-1-one, Hexan-2-one	2.498	6.605	5.565	4.764	4.531	-	<0.0001	0.02	-	1.724
102.026			0.009	0.005	0.008	0.006	0.006	-	<0.0001	<0.0001	-	0.002
103.048			0.025	0.013	0.021	0.016	0.018	-	<0.0001	<0.001	0.03	0.005
105.041			0.012	0.007	0.011	0.011	0.012	<0.0001	<0.0001	-	-	0.002
105.069	C_8_H_9_^+^	Styrene	0.047	0.017	0.029	0.021	0.022	-	<0.0001	-	-	0.019
106.079			0.021	0.015	0.024	0.014	0.017	-	-	0.01	-	0.022
107.056			0.088	0.057	0.088	0.085	0.094	<0.0001	<0.0001	-	0.05	0.018
107.086	C_8_H_11_^+^	Xylene	0.638	0.518	0.854	0.422	0.580	-	-	0.01	-	0.983
109.076	C_6_H_8_N_2_H^+^	2,5-Dimethylpyrazine	0.021	0.016	0.031	0.021	0.025	<0.001	-	0.01	-	0.017
109.103	C_8_H_13_^+^	Octenal, common fragment	0.058	0.047	0.051	0.044	0.046	<0.0001	<0.0001	<0.001	-	0.011
110.969			0.006	0.003	0.004	0.004	0.005	-	<0.0001	-	-	0.001
111.118	C_8_H_15_^+^	Octenol, Octanal	0.124	0.159	0.122	0.101	0.110	<0.0001	<0.001	0.03	-	0.028
115.079	C_6_H_10_O_2_H^+^	Caprolactone	0.031	0.018	0.027	0.022	0.023	-	<0.0001	<0.0001	-	0.005
115.113	C_7_H_14_OH^+^	Heptanal, Heptan-2-one	0.090	0.096	0.091	0.080	0.100	-	-	-	<0.001	0.022
117.092	C_6_H_12_O_2_H^+^	Hexanoic acid, Ethyl butanoate, Methyl isovalerate and other C6 esters/acids	0.030	0.017	0.025	0.023	0.023	-	<0.0001	-	-	0.005
118.056			0.002	0.001	0.002	0.003	0.003	<0.0001	<0.0001	<0.0001	0.01	0.001
119.105	C_6_H_14_O_2_H^+^		0.126	0.258	0.239	0.197	0.182	-	<0.0001	<0.001	-	0.058
121.066	C_8_H_8_OH^+^	Acetophenone, 4-Methyl-benzaldehyde	0.037	0.018	0.030	0.024	0.026	-	<0.0001	0.01	-	0.010
121.105	C_9_H_13_^+^	Trimethylbenzene	0.010	0.008	0.011	0.008	0.008	-	<0.0001	<0.0001	-	0.003
123.050	C_4_H_10_O_2_SH^+^		0.019	0.010	0.017	0.013	0.014	-	<0.0001	<0.0001	-	0.004
123.114	C_9_H_15_^+^	Nonenal	0.015	0.012	0.014	0.011	0.013	-	<0.001	<0.001	0.02	0.003
125.024	C_6_H_4_O_3_H^+^	Hydroxy-benzoquinone	0.009	0.005	0.007	0.006	0.006	-	<0.0001	<0.0001	-	0.002
125.067			0.009	0.008	0.010	0.008	0.009	-	<0.001	<0.0001	-	0.002
125.097	C_8_H_12_OH^+^	Octadienone	0.057	0.156	0.127	0.130	0.125	0.02	<0.0001	-	-	0.044
125.132	C_9_H_17_^+^	Nonanal, Nonenol	0.027	0.031	0.027	0.022	0.026	0.01	-	-	-	0.007
127.081			0.006	0.004	0.006	0.005	0.005	-	<0.0001	<0.0001	-	0.001
127.113	C_8_H_14_OH^+^	Octenal, 1-Octen-3-one	0.022	0.022	0.024	0.021	0.020	-	-	<0.001	-	0.004
128.973			0.001	0.001	0.001	0.001	0.001	<0.0001	<0.0001	<0.0001	<0.001	0.000
129.093	C_7_H_12_O_2_H^+^	Butyl propenoate, Allyl butyrate	0.014	0.008	0.012	0.010	0.011	-	<0.0001	<0.001	-	0.002
129.128	C_8_H_16_OH^+^	Octanal, Octanone	0.071	0.073	0.054	0.044	0.057	<0.0001	-	-	<0.001	0.013
130.041			0.006	0.003	0.005	0.004	0.004	-	<0.0001	<0.001	-	0.001
131.076			0.005	0.003	0.004	0.005	0.004	<0.0001	<0.0001	-	0.01	0.001
131.109	C_7_H_14_O_2_H^+^	Heptanoic acid, Ethyl-2-methylbutanoate, Ethyl-3-methylbutanoate, Methylbutyl acetate or other C7 esters/acids	0.006	0.004	0.005	0.004	0.005	-	<0.0001	<0.001	<0.001	0.001
133.112			0.006	0.005	0.005	0.005	0.006	-	<0.0001	-	<0.001	0.001
134.975			0.001	0.001	0.001	0.001	0.001	-	-	<0.001	-	0.000
135.043			0.002	0.001	0.002	0.001	0.002	-	<0.0001	<0.001	0.04	0.000
135.087	C_6_H_14_OSH^+^	3-Mercaptohexanol	0.005	0.003	0.004	0.003	0.004	-	<0.0001	<0.0001	-	0.001
137.067			0.007	0.004	0.006	0.005	0.005	-	<0.0001	<0.0001	-	0.001
137.132	C_10_H_17_^+^	Monoterpenes	0.028	0.017	0.023	0.016	0.017	0.01	<0.0001	<0.001	-	0.009
139.114	C_9_H_14_OH^+^	2,6-Nonaienal, Isophorone, Pentylfuran	0.021	0.056	0.034	0.038	0.031	0.05	<0.0001	-	0.03	0.010
141.130	C_9_H_16_OH^+^	Nonenal, Nonenone	0.006	0.008	0.007	0.006	0.007	0.01	<0.0001	-	-	0.001
143.106	C_8_H_14_O_2_H^+^	Hexenyl acetate,	0.138	0.407	0.358	0.375	0.359	<0.001	<0.0001	-	-	0.142
143.146	C_9_H_18_OH^+^	Methyloctanol, Nonanal, Nonan-2-one	0.073	0.081	0.073	0.059	0.072	0.04	-	-	-	0.021
145.060			0.002	0.001	0.002	0.002	0.002	<0.001	<0.0001	0.01	-	0.000
147.130			0.005	0.005	0.005	0.005	0.005	0.02	-	-	-	0.001
151.120			0.005	0.003	0.005	0.003	0.004	<0.001	<0.0001	<0.0001	-	0.001
153.131			0.005	0.004	0.005	0.004	0.004	-	0.02	<0.0001	-	0.001
159.137	C_9_H_18_O_2_H^+^	Nonanoic acid, 3-Methylbutyl butanoate or other C9 easters/acids	0.009	0.014	0.009	0.012	0.012	-	<0.0001	<0.0001	-	0.004
160.899			0.002	0.000	0.001	0.001	0.001	<0.0001	<0.0001	<0.001	-	0.001
161.120			0.004	0.006	0.006	0.005	0.005	0.02	<0.0001	0.01	-	0.001
165.161	C_12_H_21_^+^	2-Dodecenal	0.001	0.001	0.001	0.001	0.001	0.01	-	0.01	-	0.000
173.148			0.002	0.002	0.002	0.001	0.002	<0.001	0.02	-	0.01	0.000
175.122			0.002	0.003	0.003	0.002	0.002	0.01	<0.0001	<0.0001	-	0.000
187.169	C_11_H_22_O_2_H^+^	Methyl caprate, Ethyl nonanoate or other C11 esters/acids	0.001	0.002	0.001	0.001	0.001	0.04	<0.0001	-	-	0.001
201.182	C_11_H_24_O_2_H^+^		0.002	0.008	0.005	0.005	0.005	-	<0.0001	-	-	0.003
241.959			0.003	0.003	0.004	0.003	0.003	0.01	<0.001	<0.001	-	0.001

^1^ C = chicken; T = turkey; P = pork; B = beef; V = veal. ^2^ RMSE: root mean square error.

**Table 3:** Effects of meat species and category on the least square means and contrast significance of the scores of the latent explanatory factors (LEF) of volatile organic compounds’ proportions in cooked meat patties (in bold the correlation coefficient higher than 0.5 or lower than −0.5).

Latent Factors	Least Squares Means ^1^					Orthogonal Contrasts (*p* Value)				RMSE ^2^
	C	T	P	V	B	C + T vs. P+ V + B	C vs. T	P vs. V + B	V vs. B	%
LEF-1	0.72	−0.62	0.44	−0.40	−0.15	-	<0.001	<0.001	-	0.63
LEF-2	−0.62	0.30	0.44	−0.01	−0.12	0.03	<0.001	0.002	-	0.72
LEF-3	0.00	−0.26	0.21	−0.11	0.10	-	-	-	-	0.60
LEF-4	−0.64	−0.78	−0.30	1.15	0.54	<0.001	-	<0.001	<0.001	0.41
LEF-5	−0.16	0.09	0.10	−0.09	0.07	-	-	-	-	0.95
LEF-6	0.50	0.19	−0.46	−0.46	0.19	<0.001	-	-	0.001	0.62
LEF-7	0.79	−0.26	−0.16	−0.10	−0.25	<0.001	<0.001	-	-	0.74
LEF-8	−0.10	−0.02	0.39	−0.24	−0.03	-	-	<0.001	-	0.96
LEF-9	0.25	0.01	−0.11	−0.05	−0.11	-	-	-	-	0.44
LEF-10	0.34	0.00	−0.84	0.04	0.49	0.04	-	<0.001	0.03	0.39
LEF-11	0.18	0.40	−0.57	−0.13	0.10	<0.001	-	<0.001	-	0.69
LEF-12	0.28	0.21	−0.20	0.14	−0.43	<0.001	-	-	0.003	0.87
LEF-13	0.63	−0.43	−0.47	0.11	0.18	-	<0.001	<0.001	-	0.65
LEF-14	−0.15	−0.44	0.30	−0.31	0.58	<0.001	-	-	<0.001	0.73
LEF-15	−0.47	0.65	−0.20	−0.20	0.24	-	<0.001	-	0.007	0.69
LEF-16	0.06	−0.10	0.37	−0.13	−0.21	-	-	<0.001	-	0.89
LEF-17	0.19	0.27	−0.13	−0.03	−0.28	<0.001	-	-	-	0.68

^1^ C = chicken; T = turkey; P = pork; B = beef; V = veal. ^2^ RMSE: root mean square error.
